# Effects of Cold Stress on the Morphology and Thermogenic Capacity of Adipose Tissue in Various Anatomical Depots of *Min pigs*

**DOI:** 10.3390/cells15181661

**Published:** 2026-09-15

**Authors:** Shuo Yang, Linlin Mu, Xinmiao He, Wentao Wang, Haifeng Zhang, Ming Tian, Saihui Wu, Ziguang Liu, Heshu Chen, Siyu Ning, Xiuqin Yang, Di Liu

**Affiliations:** 1 Key Laboratory of Combining Farming and Animal Husbandry, Ministry of Agriculture and Rural Affairs, Heilongjiang Academy of Agricultural Sciences, Harbin 150086, China; hqyangshuo@foxmail.com (S.Y.);; 2 Institute of Forage and Grassland Sciences, Heilongjiang Academy of Agricultural Sciences, Harbin 150086, China; 3 College of Animal Science and Veterinary Medicine, Heilongjiang Bayi Agricultural University, Daqing 163319, China; 4 College of Animal Science and Technology, Northeast Agricultural University, Harbin 150030, China

**Keywords:** *Min pigs*, cold exposure, morphology, thermogenic

## Abstract

Cold exposure is a major physiological stimulus that promotes adipose tissue browning and enhances thermogenic capacity. As a cold-tolerant breed, *Min pigs* may rely on distinct adaptive mechanisms in adipose tissues, but how different adipose depots respond to cold stress remains unclear. This study systematically examines how cold exposure affects adipose tissue morphology, thermogenic gene expression, and the differentiation potential of preadipocytes derived from four adipose depots—cervical, axillary, perirenal, and inguinal—in *Min pigs*. Adipose tissues from the four depots were collected and analyzed using infrared thermography, histological staining, scanning electron microscopy, real-time quantitative PCR, Western blotting and in vitro differentiation assays. The results demonstrated that cold stress induced depot-specific remodeling patterns. Infrared thermography showed that the subcutaneous adipose depots including inguinal and axillary depots maintained relatively higher surface temperatures following cold exposure. Histological and ultrastructural analyses revealed typical beige-like characteristics in inguinal depots, including the accumulation of small lipid droplets, whereas changes in other depots were minimal. Molecular analyses further confirmed this heterogeneity: inguinal depots exhibited the strongest beiging response, characterized by significant upregulation of key thermogenic markers (*PGC1α*, *UCP3*, and *DIO2*) and lipolytic genes (*HSL* and *ATGL*) at the mRNA and/or protein levels. In contrast, visceral adipose depots showed weak or suppressed thermogenic activation. In vitro differentiation assays demonstrated that preadipocytes isolated from inguinal depot had the strongest beiging potential, forming tightly packed small lipid droplets and markedly increasing the expression of beige adipose-related genes upon induction. In conclusion, this study demonstrates that cold stress induces distinct beige remodeling across adipose depots in *Min pigs*, with the inguinal depot exhibiting the strongest thermogenic response and differentiation potential at morphological, molecular, and cellular levels. These findings identify the inguinal depot as a key site for adaptive thermogenesis and provide novel insights into adipose tissue remodeling and environmental adaptation in large mammals.

## 1. Introduction

Adipose tissue functions not only as the primary site for energy metabolism but also as a vital endocrine organ, with roles that extend well beyond mere energy storage. In mammals, it is broadly classified into white adipose tissue (WAT) and brown/beige adipose tissue (BAT) based on their distinct morphological, functional, and molecular characteristics [1]. WAT stores excess energy in the form of triglycerides [2], whereas BAT contains abundant mitochondria and expresses uncoupling protein 1 (*UCP1*), enabling non-shivering thermogenesis that is essential for thermal regulation in small mammals and neonates [3]. Recent studies have revealed that under specific stimuli, such as cold exposure or β-adrenergic receptor agonism, certain WAT depots can undergo a browning process and give rise to beige adipocytes with thermogenic capacity [4]. This transition involves complex morphological remodeling and the programmed upregulation of thermogenic genes, including peroxisome proliferator-activated receptor gamma coactivator 1-alpha (*PGC1α*), type II iodothyronine deiodinase (*DIO2*), and members of the UCP family [5,6]. As a result, adipose browning has attracted considerable interest as a potential therapeutic strategy for combating obesity and related metabolic disorders.

Cold stress represents one of the most potent stimuli for inducing adipose browning [7]. Exposure to low ambient temperatures activates the sympathetic nervous system, leading to the release of norepinephrine, which binds to β-adrenergic receptors on adipocytes and triggers downstream signaling cascades that promote the generation and activation of beige adipocytes [8]. To date, most studies on cold-induced adipose browning have been conducted in rodent models. In pigs, however, BAT has largely been lost during evolution, leaving them more dependent on WAT for energy storage [9]. Nevertheless, studies suggest that porcine subcutaneous WAT is capable of acquiring beige-like characteristics, thereby contributing to metabolic thermogenesis—a process commonly referred to as beiging [10,11]. Beige adipocytes, which appear sporadically within WAT in response to various stimuli, can arise either through de novo differentiation of preadipocytes or through the reprogramming of mature WAT [12,13,14]. Notably, beige adipose tissue has been identified in cold-tolerant pig breeds, including Tibetan pigs and *Min pigs* [15,16], indicating that the remarkable cold resistance of Min pigs may be partly attributable to beige adipose tissue. Despite these observations, it remains unclear how cold stress differentially influences the morphology, thermogenic gene expression, and the beiging potential of precursor cells across distinct adipose depots in Min pigs, underscoring the need for systematic investigation.

Research indicates significant functional heterogeneity among different adipose depots [17]. In small mammals, subcutaneous adipose depots exhibit a higher browning potential compared to visceral adipose depots [18]. This variation is thought to arise from differences in local microenvironment, sympathetic innervation density, vascularization, and intrinsic cellular properties [19]. In pigs, adipose depots also differ in growth characteristics, fatty acid composition, and metabolic activity [20,21]. Whether these depots display similar specificity in their response to cold stress, especially in terms of morphological remodeling and molecular changes directly linked to thermogenic function, remains an open question that warrants in-depth investigation. Addressing this issue will not only improve our understanding of adaptive thermogenesis in large animals but may also provide a theoretical foundation for enhancing cold tolerance and optimizing carcass fat distribution in pigs through nutritional or environmental modulation.

Given these gaps, the present study employed Min pigs as a model to systematically examine the effects of cold stress on adipose tissue from four different depots: cervical, axillary, perirenal and inguinal. This study is expected to characterize depot-specific thermogenic responses, identify adipose depots with high beiging potential, and provide new insights into adipose tissue remodeling and environmental adaptation mechanisms in Min pigs.

## 2. Materials and Methods

### 2.1. Animals and Experimental Design

Ten 30-day-old *Min pigs* from the same litter, six males and four females, with an average body weight of 4.0 ± 0.5 kg, were selected and randomly assigned to two groups of five each, with three males and two females per group: a room-temperature control group (RT) and a cold-stress group (CS). After one day of acclimatization at 25 °C, the CS group was exposed to 4 °C, whereas the RT group was maintained at 25 °C for the following three days. During the experimental period, all pigs were individually housed in metabolic cages with free access to water and fed a wet mash diet (feed:water = 1:1) at a daily ration of 0.25 kg per pig.

### 2.2. Sample Collection

All pigs were euthanized by intravenous injection of sodium pentobarbital at 100 mg/kg body weight prior to tissue collection. Adipose tissues were then collected from the cervical, axillary, perirenal, and left inguinal depots. A portion of each sample was fixed in 4% paraformaldehyde (BL539A, Biosharp, Beijing, China) for hematoxylin and eosin (H&E) staining. Another portion was fixed in 2.5% glutaraldehyde (DF0156, Leagene, Beijing, China) for scanning electron microscopy (SEM) analysis. The remaining tissue samples were immediately snap-frozen in liquid nitrogen for subsequent RNA and protein extraction.

### 2.3. Infrared Thermography

All infrared thermographic images were acquired using a FLUKE handheld infrared camera (Ti100, FLUKE, Everett, WA, USA) with an emissivity setting of 0.92, which has a measurement range of –20 °C to 250 °C. The camera is equipped with an IR-OptiFlex^TM^ focusing system (FLUKE, Everett, WA, USA); the focus was set at a distance of 4 feet, and the images were taken from a distance of 5 feet. Images were acquired with the camera positioned directly facing the dorsal and ventral surfaces of the piglet. For quantitative analysis, five images were captured per animal, and the temperature values were averaged for each body side (dorsal and ventral) from these five measurements. To minimize stress induced by restraint, the experimenter took each piglet out of the metabolic cage, held it by the hind legs, rapidly inverted it, and immediately captured the thermal image.

### 2.4. Morphological Observation

For H&E staining, adipose tissues were fixed overnight in 4% paraformaldehyde, dehydrated through a graded ethanol series, and embedded in paraffin. At least five sections were prepared from each sample, with a total of 25 sections per group. Sections were cut at a thickness of 10 μm. Sections were stained with H&E (BL700B, biosharp, Beijing, China), and imaged using a microscope (DP40; OLYMPUS, Tokyo, Japan).

The internal structure of adipose tissue from the four depots was observed using a scanning electron microscope (SEM) (S-4800; Hitachi Ltd., Tokyo, Japan). Samples were mounted on metal stubs, sputter-coated with platinum, and observed at an accelerating voltage of 5 kV. Images were recorded using a Canon digital camera (Canon Inc., Tokyo, Japan).

### 2.5. Real-Time Quantitative PCR (RT-qPCR)

Total RNA was extracted using TRIzol reagent (420810, Ambion, Beijing, China). The RNA quality was assessed with the absorbance ratio 260/280, and only samples with ratios between 1.8 and 2.0 were used for further analysis. The extracted RNA was reverse-transcribed into cDNA using a PrimeScript^TM^ RT Reagent Kit (RR047a, Takara, Beijing, China). RT-qPCR for mRNA expression analysis was conducted using an UltraSYBR One-Step RT-qPCR Kit (01170, Cwbio, Beijing, China) with GAPDH as the internal control. The relative expression levels were calculated using the 2^−ΔΔCt^ method. Each sample was run in triplicate. Primer sequences are provided in Table S1.

### 2.6. Western Blotting

Total protein was extracted from adipose tissue using RIPA lysis buffer (R0010, Solarbio, Beijing, China), and protein concentration was quantified with a BCA protein assay kit (KTD3001, Abbkine, Wuhan, Hubei, China). Proteins were then separated by SDS-PAGE (P1200, Solarbio, Beijing, China) and transferred onto PVDF membranes (IPVH00010, Millipore, Ireland). Protein bands were visualized via chemiluminescence detection using a ChemStudio SA system (Analytik Jena, Jena, Germany). The primary antibodies used included: DIO2 (1:1000, ER1907-46, HUABIO, Hangzhou, Zhejiang, China), CEBPα (1:1000, ET1612-46, HUABIO, Hangzhou, Zhejiang, China), FABP4 (1:5000, ET1703-98, HUABIO, Hangzhou, Zhejiang, China), ATGL (1:1500, HA500301, HUABIO, Hangzhou, Zhejiang, China), PPARγ (1:1000, YA122, MCE, Shanghai, China), β-Tubulin (1:10,000, 100109-MCR05, Sino Biological, Beijing, China), PGC1α (1:1000, bsm-61390R, BIOSS, Beijing, China), LPL (1:1000, A16252, Abclonal, Wuhan, Hubei, China), UCP3 (1:1000, A16995, Abclonal, Wuhan, Hubei, China), and HSL (1:50,000, A15686, Abclonal, Wuhan, Hubei, China).

### 2.7. Isolation and Culture of Porcine SVF Cells

Adipose tissues from the cervical, axillary, perirenal, and inguinal depots were collected from five one-month-old male Min pigs. After thorough dissection, the tissues were minced into small pieces and digested at 37 °C for 90 min. Following centrifugation and filtration of the tissue suspension, the pelleted stromal vascular fraction (SVF) cells were collected and suspended in DMEM/F12 medium (PM150312, Pricella, Wuhan, Hubei, China).

When SVF cells reached 100% confluence, adipogenic differentiation was induced. To generate beige adipocytes, the cells were first incubated in a beige induction medium composed of DMEM/F12 supplemented with 10% fetal bovine serum (FBS, 209111, NEST, Wuxi, Jiangsu, China), 0.5 mM 3-isobutyl-1-methylxanthine (IBMX), 1 μM dexamethasone, 10 μg/mL insulin, 1 μM rosiglitazone, 1 μM triiodothyronine (T3), and 1 μM indomethacin for 2 days. This was followed by a maintenance phase in DMEM/F12 containing 10% FBS, 10 μg/mL insulin, 1 μM T3, and 1 μM rosiglitazone for an additional 6 days, with the medium refreshed every 2 days. To generate white adipocytes, the cells were induced using a white induction medium consisting of DMEM/F12 supplemented with 10% FBS, 0.5 mM IBMX, 1 μM dexamethasone, 10 μg/mL insulin, and 1 μM indomethacin for 2 days, followed by maintenance in DMEM/F12 containing 10% FBS and 10 μg/mL insulin alone for an additional 6 days, with medium changes every 2 days.

### 2.8. Oil Red O Staining

On days 0, 2, 4, 6, and 8 following adipogenic induction, cells were stained using an Oil Red O staining kit (1208964, Leyan, Shanghai, China). Stained cells were imaged using an optical microscope (DP40; OLYMPUS, Tokyo, Japan).

### 2.9. Statistical Analysis

All experiments were performed with a minimum of three independent biological replicates. Data are presented as the mean ± standard error of the mean (SEM). For in vivo experiments, gene expression data were analyzed using a mixed-effects model with treatment (cold vs. room temperature), adipose depot (cervical, axillary, perirenal, and inguinal), and their interaction as fixed factors, and animal as a random factor to account for the repeated measurements from the same individuals across multiple depots. For in vitro differentiation experiments, a similar mixed-effects model was applied with differentiation condition (white vs. beige), depot origin, and their interaction as fixed factors, and biological replicate as a random factor. Post hoc pairwise comparisons were performed using Tukey’s or Bonferroni’s multiple comparison tests. To control the family-wise error rate across multiple genes, Bonferroni correction was applied within each gene category (beige marker genes, adipogenesis-related genes, and lipid metabolism-associated genes). Statistical analyses were conducted using GraphPad Prism 6.01 or SAS 9.4. Differences were considered statistically significant at *p* < 0.05 (adjusted where applicable) (* *p* < 0.05, ** *p* < 0.01, *** *p* < 0.001, **** *p* < 0.0001).

## 3. Results

### 3.1. Morphological Characteristics of Adipose Tissue Under Cold Stress

**Figure 1:**
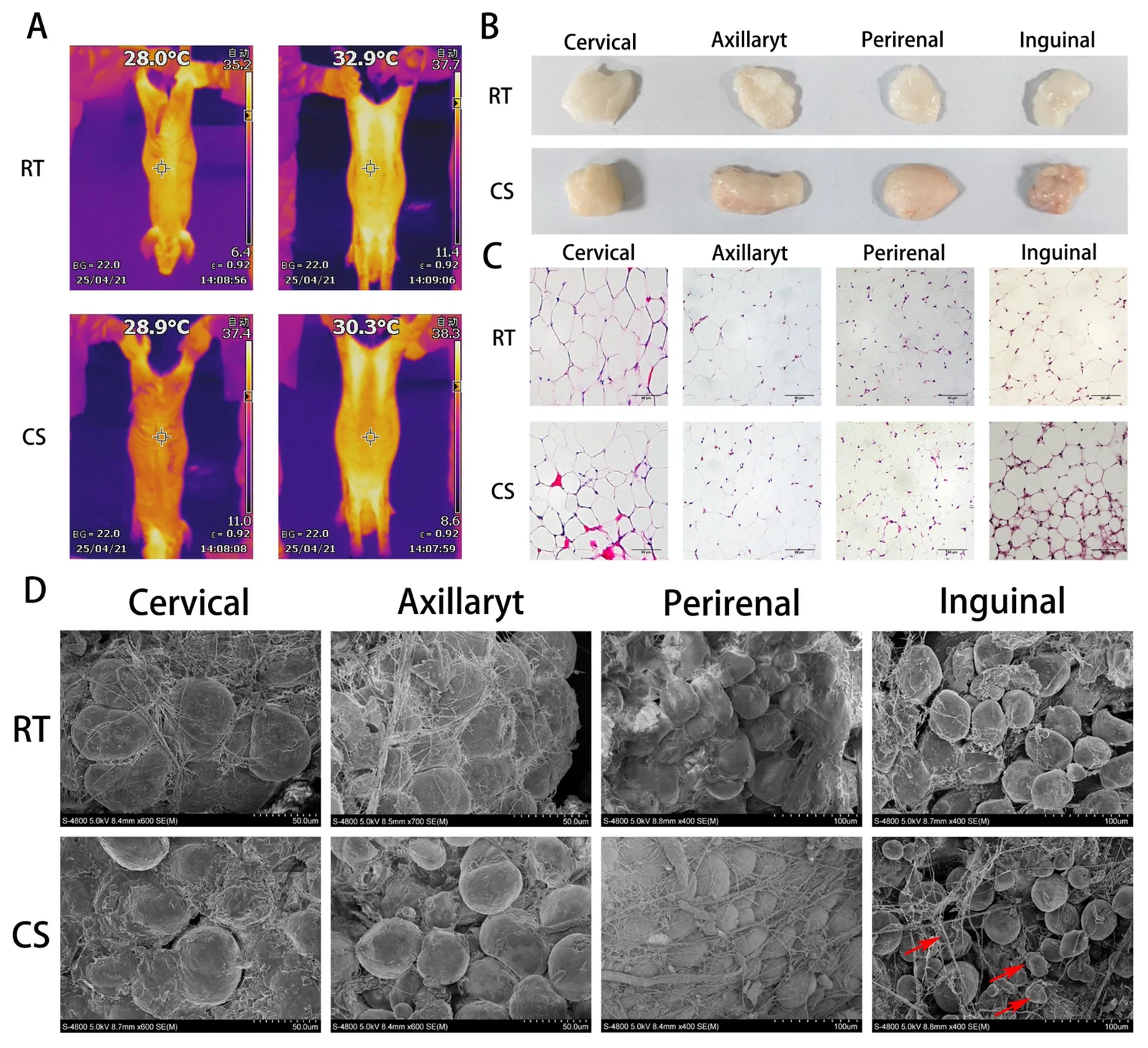
Effects of cold stress on morphological characteristics of adipose tissue. (**A**) Infrared thermography (The crosshair indicates the temperature detection point). (**B**) Representative images of adipose tissue. (**C**) H&E staining images of adipose tissue (scale bar = 50 µm). (**D**) SEM images of adipose tissue (Red arrows indicate multilocular lipid droplets).

The infrared thermography results revealed distinct differences in body surface temperature between the CS group and RT group. In the RT group, the ventral and dorsal temperatures averaged 29.54 °C and 32.64 °C, respectively, whereas the corresponding temperatures in the CS group were 29.44 °C and 29.36 °C. Notably, the inguinal and axillary depots consistently maintained relatively higher temperatures in both groups (Figure 1A). Representative adipose samples collected from the cervical, axillary, perirenal, and inguinal depots are shown in Figure 1B. Compared to the RT group, the adipose tissues from the CS group exhibited marked alterations in both the color and macroscopic morphology, indicating that cold stress induced macroscopic morphological remodeling of adipose tissues. Histological staining was performed to analyze the cellular structure of adipose tissues from the four depots (Figure 1C). In response to cold stress, the inguinal depot developed typical morphological features of BAT, including numerous small lipid droplets and a tightly packed cellular structure. Ultrastructural analysis via SEM (Figure 1D) further demonstrated that cold stress induced evident lipid droplet fission in the inguinal depot.

### 3.2. Effects of Cold Stress on the Expression of Thermogenesis-Related Genes in Adipose Tissues from Different Depots

To investigate the influence of cold stress on the thermogenic capacity of adipose tissues from distinct depots, the expression of thermogenesis-related genes was analyzed at both the mRNA and protein levels. These genes were categorized into beige adipocyte marker genes (*PGC1α*, *UCP3*, *DIO2*, *CIDEA*, *COX7A1*, *EBF2*, and *PDK4*), adipogenesis-related genes (*PPARγ*, *C/EBPα*, and *FASN*), lipid synthesis/metabolism-related genes (*LPL*, *FASN*, and *FABP4*), and lipolysis-related genes (*HSL* and *ATGL*), with results detailed below.

**Figure 2:**
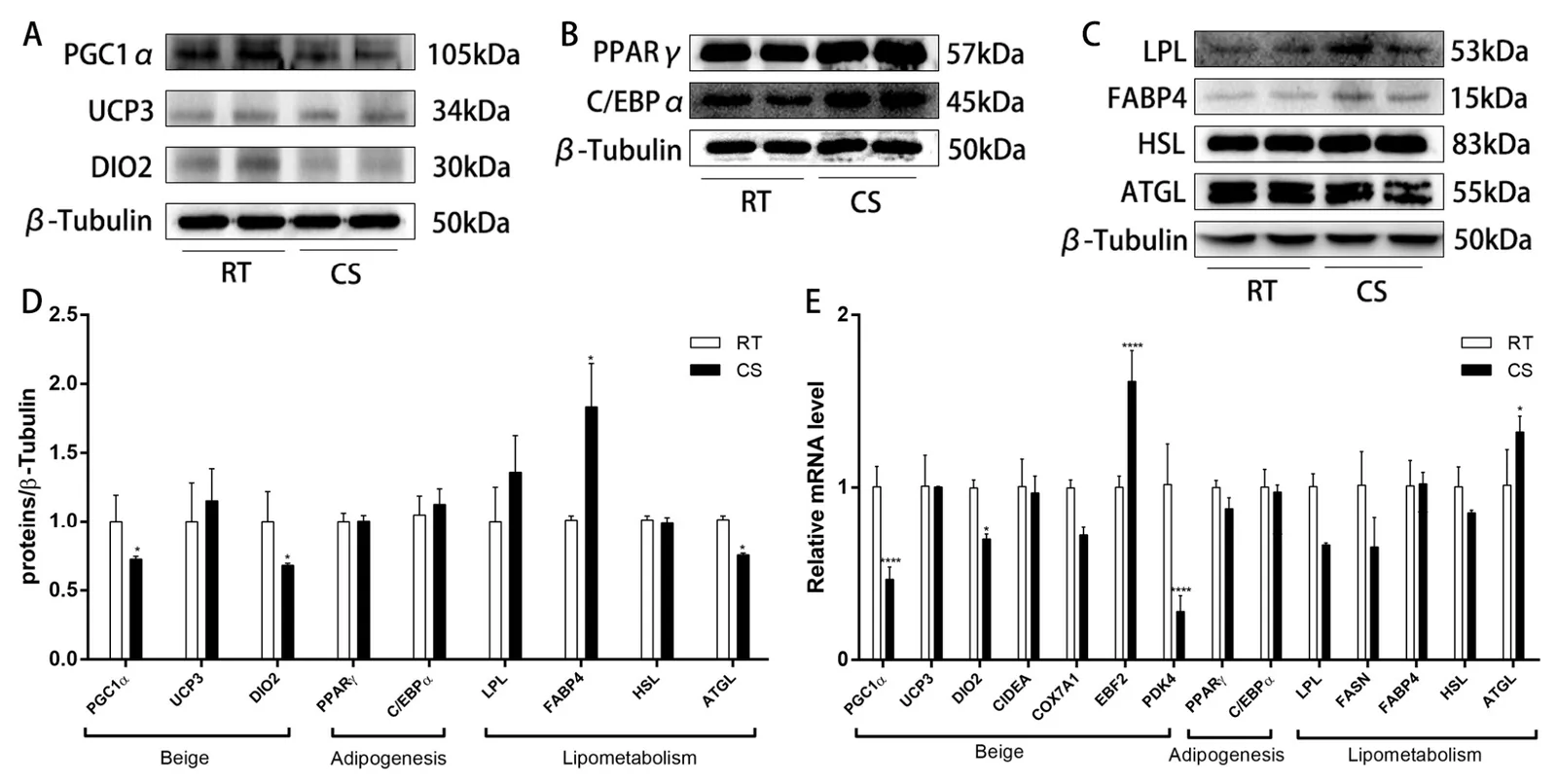
Effects of cold stress on the expression of thermogenesis-related genes in cervical adipose tissue. (**A**) Protein expression levels of beige adipocyte marker genes. (**B**) Protein expression levels of adipogenesis-related genes. (**C**) Protein expression levels of lipid metabolism-associated genes. (**D**) Quantitative analysis of grayscale values. (**E**) mRNA expression levels of beige adipocyte marker genes, adipogenesis-related genes, and lipid metabolism-associated genes (* *p* < 0.05, **** *p* < 0.0001). Statistical significance was determined by a mixed-effects model with Bonferroni correction for multiple comparisons.

For the cervical depot (Figure 2), protein-level analysis revealed that, compared with the RT group, cold stress significantly decreased the expression of beige adipocyte marker genes *PGC1α* and *DIO2*, significantly increased the expression of the fatty acid metabolism-related gene *FABP4*, and significantly reduced the expression of the lipolysis-related gene *ATGL* (all *p* < 0.05). No significant differences were observed in the protein levels of the remaining genes (*p* > 0.05). At the mRNA level, the CS group exhibited highly significant downregulation of the beige adipocyte marker genes *PGC1α* and *PDK4*, as well as the lipid synthesis-related genes *LPL* and *FASN* (all *p* < 0.01). In addition, *DIO2* was significantly downregulated (*p* < 0.05), whereas the beige marker gene *EBF2* was highly upregulated (*p* < 0.01). The lipolysis-related gene *ATGL* also showed significant upregulation (*p* < 0.05), while no significant changes were detected in the remaining transcripts (*p* > 0.05).

**Figure 3:**
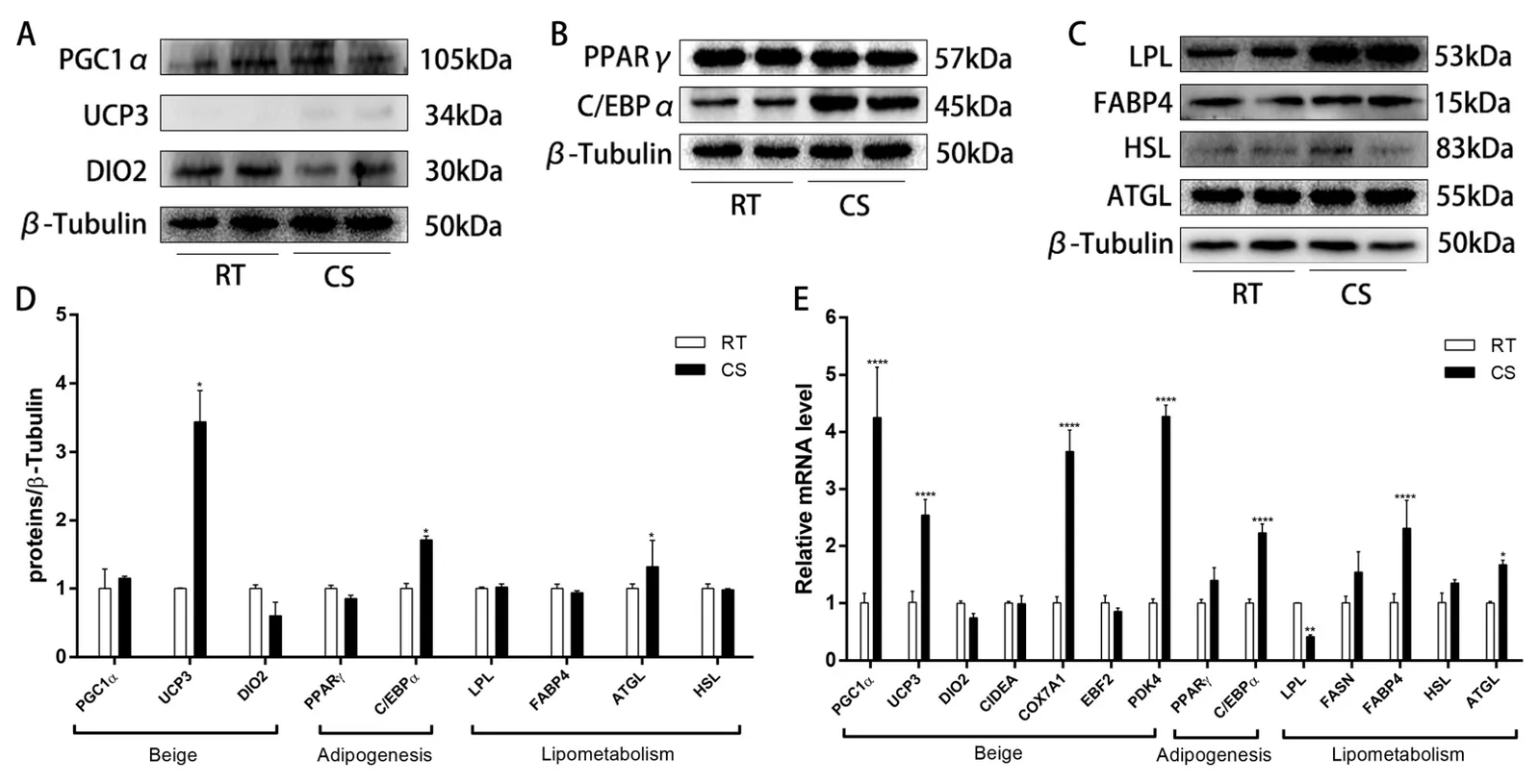
Effects of cold stress on the expression of thermogenesis-related genes in axillary adipose tissue. (**A**) Protein expression levels of beige adipocyte marker genes. (**B**) Protein expression levels of adipogenesis-related genes. (**C**) Protein expression levels of lipid metabolism-associated genes. (**D**) Quantitative analysis of grayscale values. (**E**) mRNA expression levels of beige adipocyte marker genes, adipogenesis-related genes, and lipid metabolism-associated genes (* *p* < 0.05, ** *p* < 0.01, **** *p* < 0.0001). Statistical significance was determined by a mixed-effects model with Bonferroni correction for multiple comparisons.

In the axillary depot (Figure 3), protein-level analysis revealed that, compared with the RT group, the CS group exhibited significantly higher expression of the beige adipocyte marker gene *UCP3*, the adipogenesis-related gene *C/EBPα*, and the lipolysis-related gene *ATGL* (all *p* < 0.05). No significant differences were observed in the protein expression of other genes (*p* > 0.05). At the mRNA level, all beige adipocyte marker genes, with the exception of *DIO2*, *CIDEA*, and *EBF2*, were significantly upregulated in the CS group (*p* < 0.01). In contrast, the lipid synthesis-related gene *LPL* was markedly downregulated (*p* < 0.01). Furthermore, the adipogenesis-related gene *C/EBPα*, the fatty acid metabolism-related gene *FABP4*, and the lipolysis-related gene *ATGL* showed significant or highly significant upregulation (*C/EBPα*, *FABP4*: *p* < 0.01; *ATGL*: *p* < 0.05).

**Figure 4:**
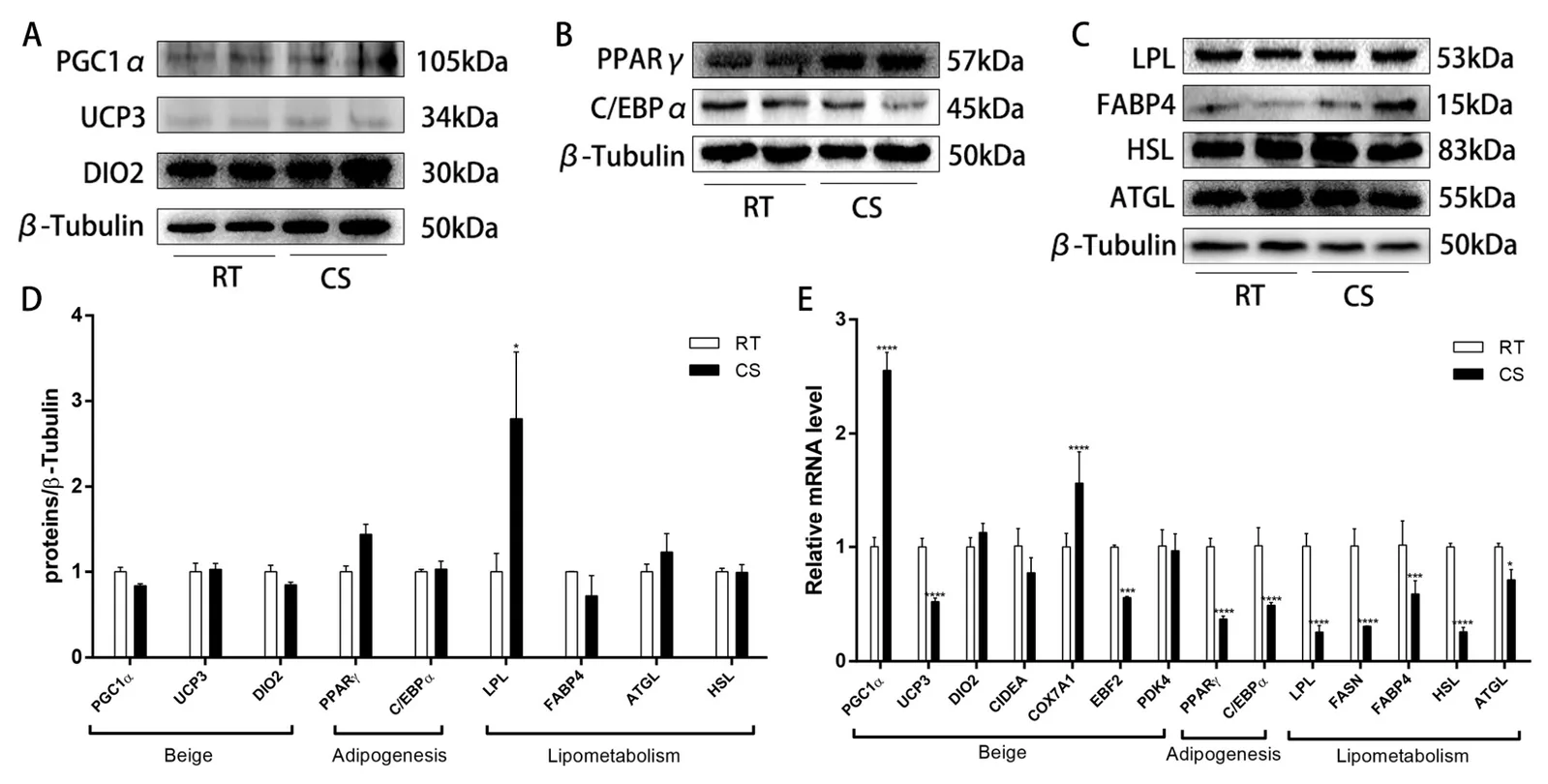
Effects of cold stress on the expression of thermogenesis-related genes in perirenal adipose tissue. (**A**) Protein expression levels of beige adipocyte marker genes. (**B**) Protein expression levels of adipogenesis-related genes. (**C**) Protein expression levels of lipid metabolism-associated genes. (**D**) Quantitative analysis of grayscale values. (**E**) mRNA expression levels of beige adipocyte marker genes, adipogenesis-related genes, and lipid metabolism-associated genes (* *p* < 0.05, *** *p* < 0.001, **** *p* < 0.0001). Statistical significance was determined by a mixed-effects model with Bonferroni correction for multiple comparisons.

For the perirenal depot (Figure 4), protein-level analysis revealed that only the lipid synthesis-related gene LPL was expressed at significantly higher levels in the CS group than in the RT group (*p* < 0.05), whereas no significant differences were observed in the protein expression of other genes (*p* > 0.05). At the mRNA level, the CS group exhibited highly significant upregulation of the beige adipocyte marker genes *PGC1α* and *COX7A1*, alongside highly significant downregulation of other beige marker genes, including *UCP3* and *EBF2* (all *p* < 0.01); notably, all adipogenesis-related genes and lipid metabolism-associated genes were highly significantly downregulated (*p* < 0.01).

**Figure 5:**
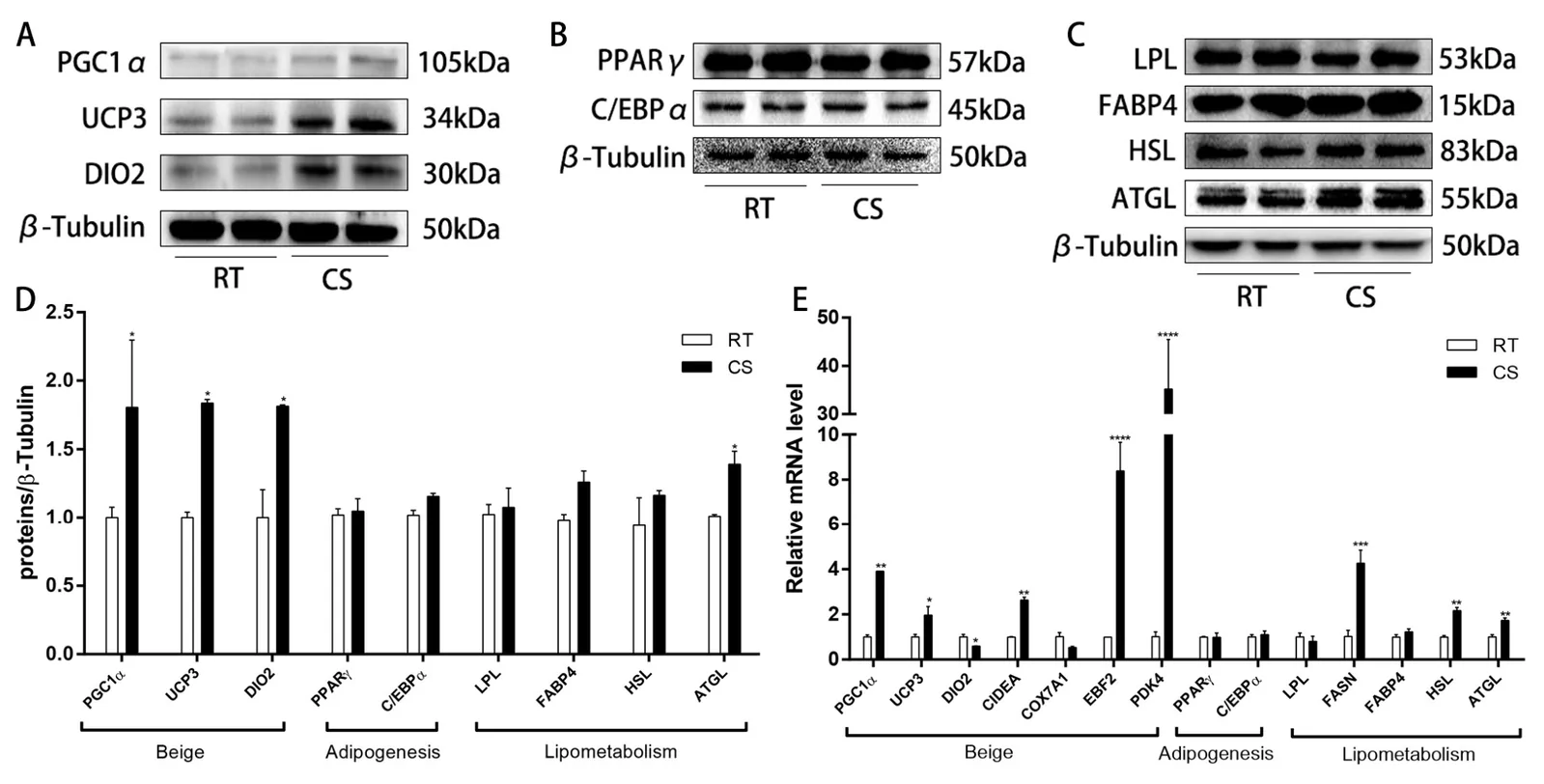
Effects of cold stress on the expression of thermogenesis-related genes in inguinal adipose tissue. (**A**) Protein expression levels of beige adipocyte marker genes. (**B**) Protein expression levels of adipogenesis genes. (**C**) Protein expression levels of lipid metabolism-associated genes. (**D**) Quantitative analysis of grayscale values. (**E**) mRNA expression levels of beige adipocyte marker genes, adipogenesis-related genes, and lipid metabolism-associated genes (* *p* < 0.05, ** *p* < 0.01, *** *p* < 0.001, **** *p* < 0.0001). Statistical significance was determined by a mixed-effects model with Bonferroni correction for multiple comparisons.

In the inguinal depot (Figure 5), protein-level analysis indicated that the CS group exhibited significantly increased expression of all detected beige adipocyte marker genes (three in total) and the lipolysis-related gene *ATGL* compared with the RT group (all *p* < 0.05). In contrast, proteins from other gene categories displayed an increasing trend, although these changes did not reach statistical significance (*p* > 0.05). At the mRNA level, the CS group displayed significant upregulation of the beige adipocyte marker gene *UCP3* (*p* < 0.05) and highly significant upregulation of other beige marker genes, including *PGC1α*, *CIDEA*, *EBF2* and *PDK4* (all *p* < 0.01), The mixed-effects model analysis further confirmed a significant treatment × depot interaction for *PGC1α*, *UCP3*, *CIDEA*, *EBF2* and *PDK4* (*p* < 0.05), indicating that the cold-induced upregulation in the inguinal depot was significantly greater than that in other depots. While no significant differences were observed in the adipogenesis-related genes *PPARγ* and *C/EBPα*, the adipogenesis-related gene *FASN* and lipolysis-related genes *HSL* and *ATGL* were highly significantly upregulated (all *p* < 0.01).

Collectively, these findings demonstrate that among the adipose tissue depots examined, inguinal adipose tissue exhibits the most prominent cold-induced browning of WAT, accompanied by enhanced lipolysis.

### 3.3. Browning Potential of Adipocytes from Different Anatomical Depots

To evaluate the browning potential of adipocytes derived from distinct anatomical depots, primary preadipocytes were isolated from various adipose tissues, cultured, and passaged to the third generation (P3) before induction of either WAT or BAT. Oil Red O staining was performed at days 0, 2, 4, 6, and 8 after induction to assess the accumulation of lipid droplets. Additionally, mRNA expression levels of beige adipocyte marker genes, adipogenesis-related genes, and lipid metabolism-associated genes were determined, and the ratio of gene expression at day 8 to day 2 (day 8/day 2) was calculated to assess the extent of preadipocyte differentiation.

**Figure 6:**
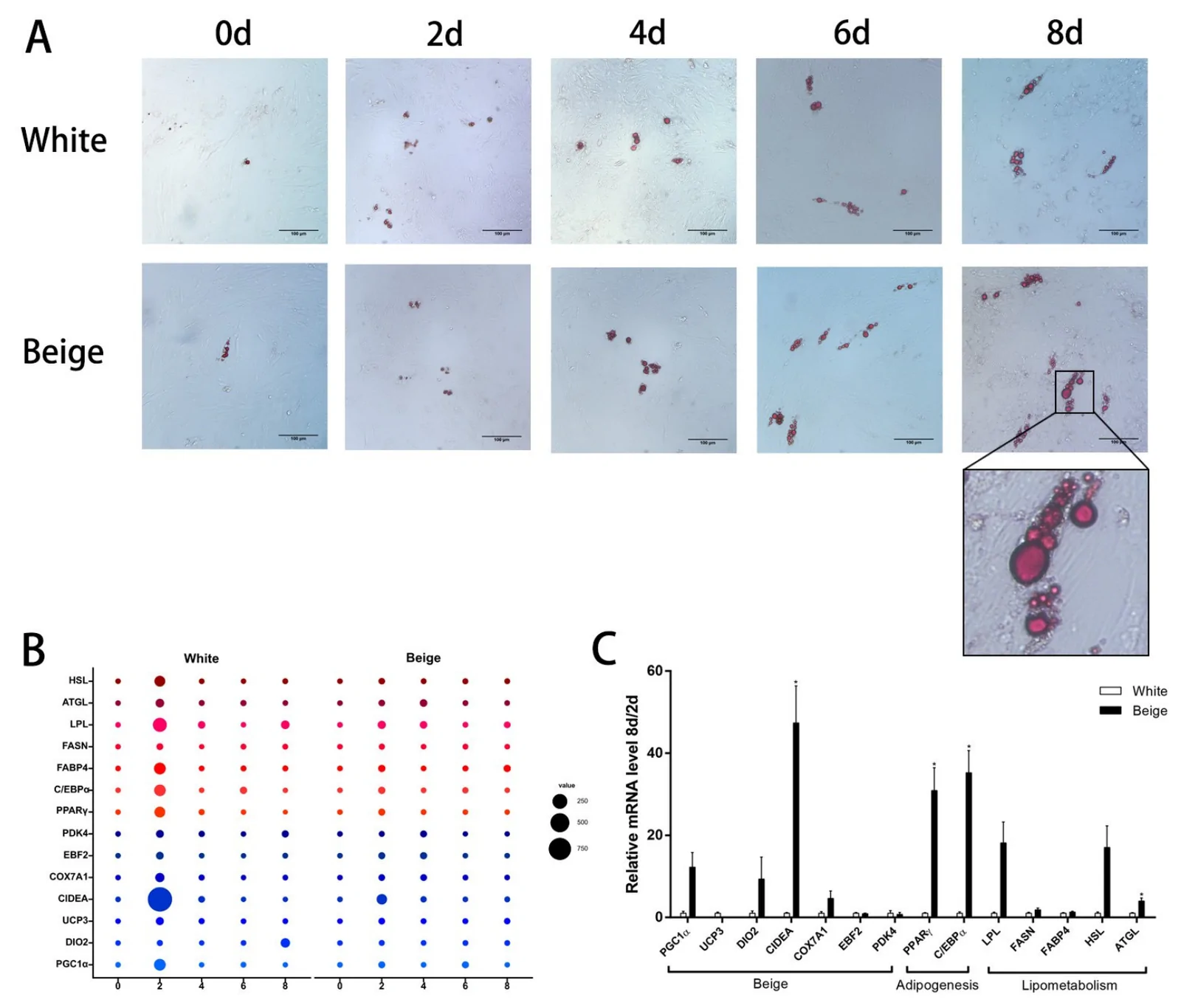
Beiging potential of cervical preadipocytes. (**A**) Oil Red O staining (scale bar = 100 µm). (**B**) mRNA expression levels of beige adipocyte marker genes, adipogenesis-related genes, and lipid metabolism-associated genes. The color of the circles represents different gene categories: blue indicates beige adipocyte marker genes, while red indicates adipogenesis and lipid metabolism-related genes. The size of the circles indicates the expression value. (**C**) Ratios of mRNA expression levels at day 8/day 2 (* *p* < 0.05). Statistical significance was determined by a mixed-effects model with Bonferroni correction for multiple comparisons.

For cervical primary preadipocytes (Figure 6), Oil Red O staining revealed a progressive increase in lipid droplet accumulation with prolonged induction time in both WAT and BAT groups, with larger lipid droplets observed in the BAT group at day 8. At the mRNA level, all examined genes in the WAT group reached a peak at day 2 and subsequently declined, whereas genes in the BAT group exhibited variable peak expression times, although a sharp increase in gene expression was consistently observed at day 2. The day 8/day 2 expression ratio indicated that the BAT group displayed significantly higher ratios of the beige marker gene *CIDEA*, adipogenesis-related genes *PPARγ* and *C/EBPα*, and the lipolysis-related gene *ATGL* compared with the WAT group (all *p* < 0.05), while no significant differences were observed for other gene categories (*p* > 0.05).

**Figure 7:**
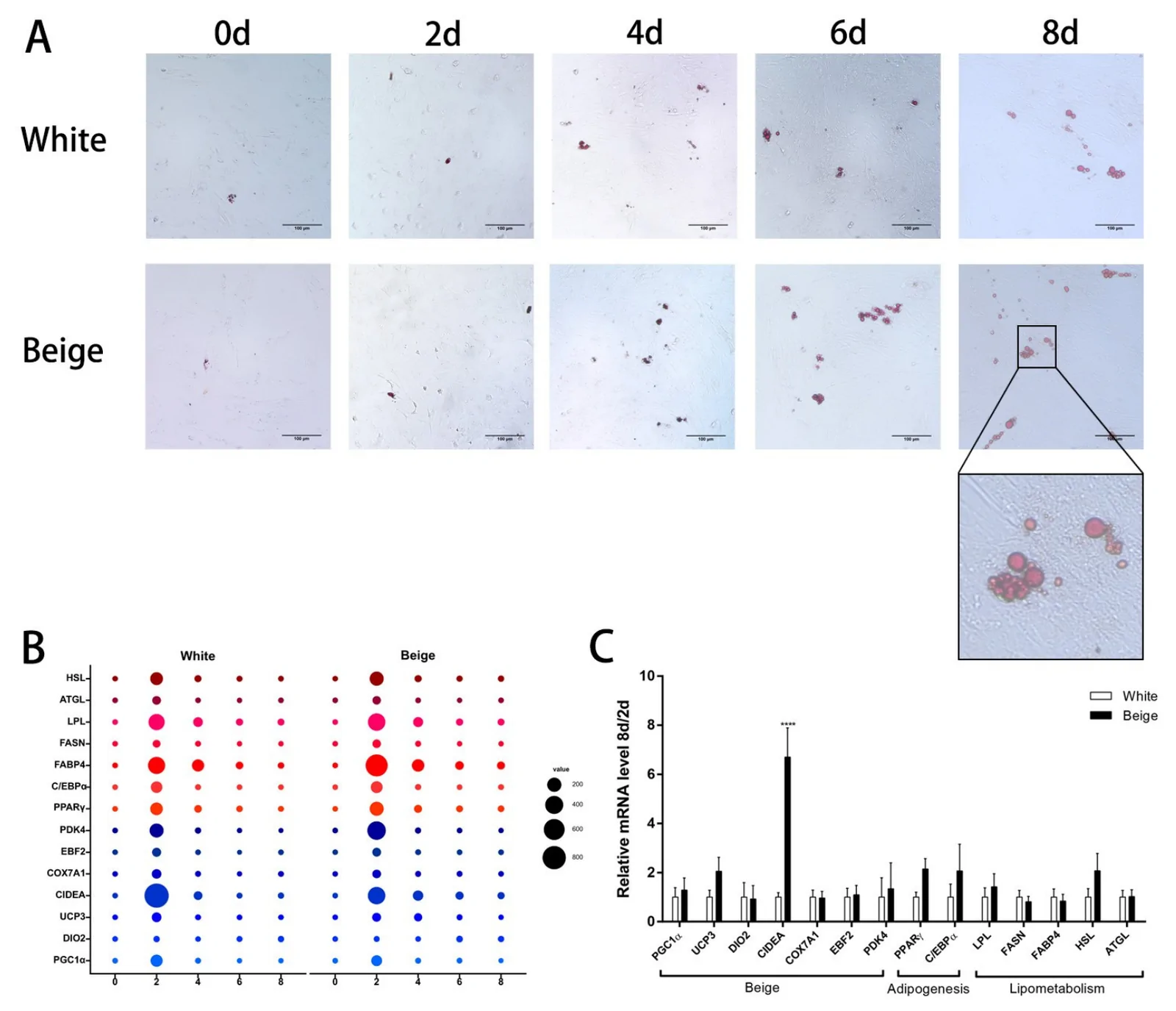
Beiging potential of axillary preadipocytes. (**A**) Oil Red O staining (scale bar = 100 µm). (**B**) mRNA expression levels of beige adipocyte marker genes, adipogenesis-related genes, and lipid metabolism-associated genes. The color of the circles represents different gene categories: blue indicates beige adipocyte marker genes, while red indicates adipogenesis and lipid metabolism-related genes. The size of the circles indicates the expression value. (**C**) Ratios of mRNA expression levels at day 8/day 2 (**** *p* < 0.0001). Statistical significance was determined by a mixed-effects model with Bonferroni correction for multiple comparisons.

For axillary primary preadipocytes (Figure 7), lipid droplet numbers increased throughout the induction period in both groups, with larger lipid droplets observed in the BAT group at day 8. All examined genes in both differentiation groups reached peak mRNA expression at day 2, followed by a decline. The day 8/day 2 ratio showed that only the beige adipocyte marker gene *CIDEA* showed a significantly higher ratio in the BAT group compared with the WAT group (*p* < 0.01), whereas no significant differences were detected for the remaining gene categories (*p* > 0.05).

**Figure 8:**
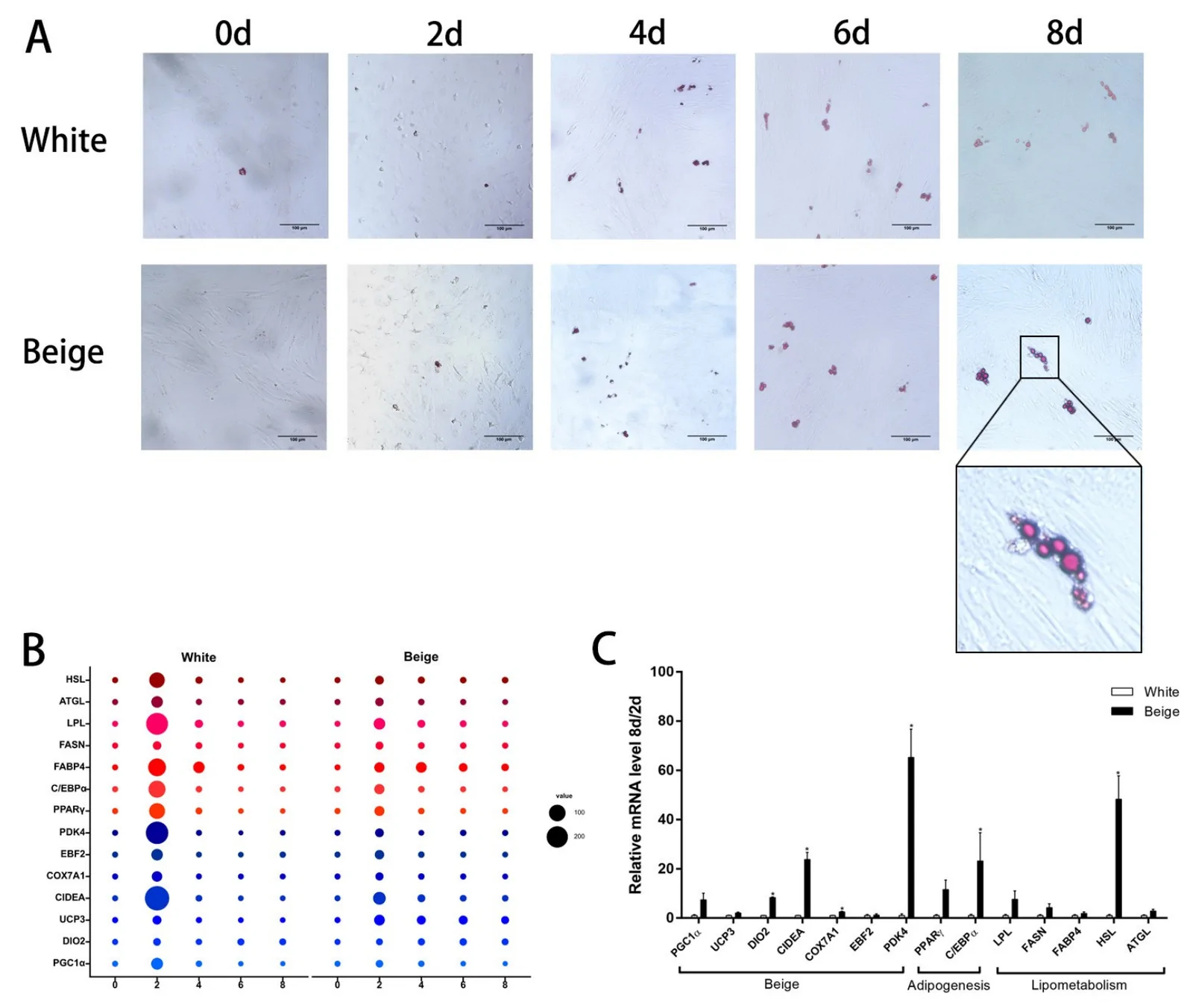
Beiging potential of perirenal preadipocytes. (**A**) Oil Red O staining (scale bar = 100 µm). (**B**) mRNA expression levels of beige adipocyte marker genes, adipogenesis-related genes, and lipid metabolism-associated genes. The color of the circles represents different gene categories: blue indicates beige adipocyte marker genes, while red indicates adipogenesis and lipid metabolism-related genes. The size of the circles indicates the expression value. (**C**) Ratios of mRNA expression levels at day 8/day 2 (* *p* < 0.05). Statistical significance was determined by a mixed-effects model with Bonferroni correction for multiple comparisons.

For perirenal primary preadipocytes (Figure 8), lipid droplet accumulation increased over time in both groups, and larger lipid droplets were again observed in the WAT group at day 8. All genes in both induction groups peaked at day 2 before declining. The day 8/day 2 expression ratio demonstrated that the BAT group exhibited significantly higher ratios of beige adipocyte marker genes *DIO2*, *CIDEA* and *PDK4*, the adipogenesis-related gene *C/EBPα*, as well as the lipolysis-related gene HSL compared with the WAT group (all *p* < 0.05).

**Figure 9:**
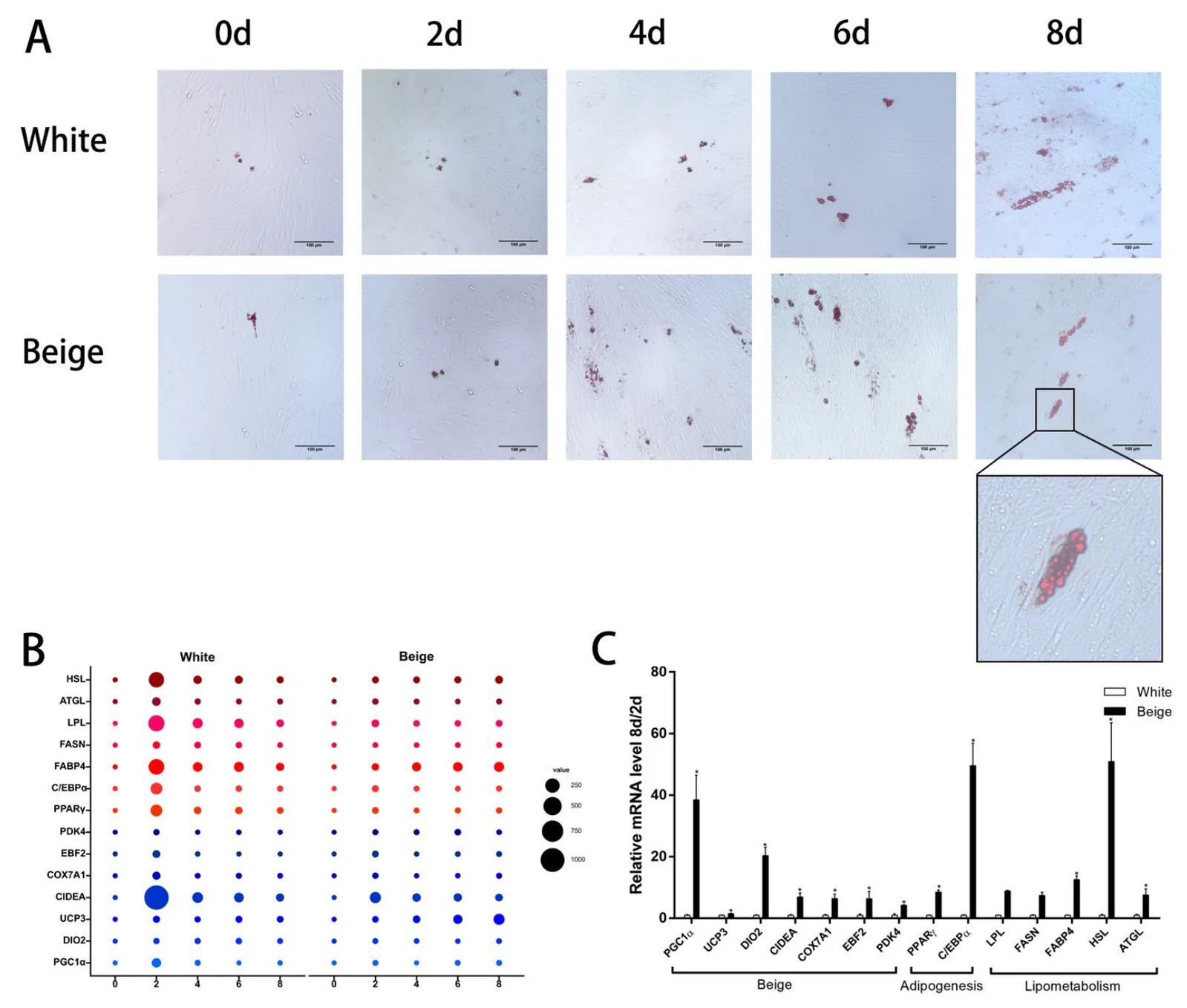
Beiging potential of inguinal preadipocytes. (**A**) Oil Red O staining (scale bar = 100 µm). (**B**) mRNA expression levels of beige adipocyte marker genes, adipogenesis-related genes, and lipid metabolism-associated genes. The color of the circles represents different gene categories: blue indicates beige adipocyte marker genes, while red indicates adipogenesis and lipid metabolism-related genes. The size of the circles indicates the expression value. (**C**) Ratios of mRNA expression levels at day 8/day 2 (* *p* < 0.05). Statistical significance was determined by a mixed-effects model with Bonferroni correction for multiple comparisons.

For inguinal primary preadipocytes (Figure 9), lipid droplet numbers increased in both groups during induction, whereas the BAT group formed tightly clustered small lipid droplets at day 8. In the WAT group, all genes peaked at day 2 and then decreased, whereas the BAT group showed gene-specific peak expression times. The day 8/day 2 ratio demonstrated that the BAT group had significantly higher ratios across all tested gene categories, including beige adipocyte marker genes, adipogenesis-related genes, and lipid metabolism-associated genes, compared with the WAT group (all *p* < 0.05).

Collectively, the combined Oil Red O staining and mRNA expression data demonstrate that inguinal primary preadipocytes exhibit the strongest potential for BAT differentiation among the four adipose depots examined.

## 4. Discussion

This study systematically revealed, for the first time, the macro- and micro-morphological remodeling of adipose tissues from four distinct anatomical depots in Min pigs in response to cold stress, using infrared thermography, histological staining, SEM, RT-qPCR and Western blotting. Previous studies on mammalian BAT have reported considerable debate regarding its exact distribution, with research focusing on depots including the inguinal, epididymal, perirenal, abdominal, axillary, and scapular regions [22,23]. With advances in imaging technologies, positron emission tomography-computed tomography (PET-CT) has emerged as a powerful tool for assessing the thermogenic activity of adipose tissue and has been widely applied in humans and rodents [24,25]. However, due to experimental and technical limitations, investigations of thermogenic adipose depots in pigs, a large mammalian species, remain relatively scarce and less precise. To date, only a limited number of studies have employed PET-CT to investigate thermogenic adipose depots in pigs. For example, Lin et al. selected Bama pigs and Tibetan pigs as research models and demonstrated that following 4 h of cold exposure, Tibetan pigs exhibited pronounced F18-fluorodeoxyglucose (18F-FDG) uptake in perirenal, axillary, and inguinal depots, whereas Bama pigs showed no detectable thermogenic signal [15].

The present study aimed to identify thermogenic adipose depots in *Min pigs* under cold stress. Infrared thermography revealed that the inguinal and axillary depots consistently maintained relatively high temperatures during cold exposure, consistent with the functional characteristics of adipose tissues involved in non-shivering thermogenesis [26]. Macroscopic morphological observations further revealed that axillary and inguinal depots exhibited the most significant changes in color and structure following cold stress. Histological analysis confirmed that the inguinal depot displayed typical features of BAT, including the accumulation of small lipid droplets and a tightly packed cellular structure. Moreover, SEM analysis revealed evident lipid droplet fragmentation in this depot, indicating structural remodeling associated with white-to-beige adipocyte conversion. These findings are consistent with previous reports showing beige-like characteristics in subcutaneous adipose tissue of *Tibetan pigs* following cold exposure [27], supporting the idea that specific adipose depots in cold-tolerant pig breeds possess cold-induced morphological remodeling. Notably, adipose tissues from different depots exhibited significant heterogeneity in their morphological responses to cold stress: cervical and perirenal depots showed no obvious conversion to small lipid droplets, whereas inguinal and axillary depots underwent prominent morphological remodeling. This difference partially aligns with the classical concept in small mammals that subcutaneous adipose tissue has greater browning potential than visceral adipose tissue [28]. However, discrepancies exist among pig studies. Lin et al. reported larger adipocytes in perirenal, greater omental, and inguinal depots compared with smaller subcutaneous back adipocytes, a finding that differs from the present study [17]. The current research indicates a more complex, depot-specific pattern in pigs: the inguinal depot exhibited the strongest remodeling response, followed by the axillary depot, whereas the perirenal depot displayed minimal beige-like morphological characteristics. This species- and depot-specific difference may reflect the unique evolutionary trajectory of pigs, which are thought to have lost typical BAT during evolution, leading to compensatory thermogenic differentiation within selected subcutaneous adipose depots [29]. Differences in local microenvironments may further amplify this heterogeneity. The sustained elevation in surface temperature of the inguinal and axillary depots, as revealed by infrared thermography, further supports the physiological role of adipose tissue in these depots in initiating thermogenic activity through morphological remodeling, thereby supporting the maintenance of thermal balance under cold stress.

The programmed upregulation of thermogenic gene expression represents a core molecular hallmark of adipose tissue beiging [30]. In the present study, analyses at both the mRNA and protein levels revealed pronounced depot-specific differences in the molecular responses of adipose tissues from *Min pigs* to cold stress. Among the depots examined, the inguinal depot exhibited the most significant activation of thermogenic gene expression. At the mRNA level, key beige marker genes such as *PGC1α*, *UCP3*, and *CIDEA* were extremely significantly upregulated, and corresponding protein levels were also significantly increased. Meanwhile, ATGL was significantly upregulated at both the mRNA and protein levels, whereas HSL expression was increased only at the mRNA level. As a core regulatory factor of mitochondrial biogenesis and thermogenic pathways, the high expression of *PGC1α* directly drives the transcriptional activation of UCP family genes [31]. *UCP3*, a key effector of adipose tissue thermogenesis, promotes the conversion of chemical energy into thermal energy when its expression is elevated [32]. The coordinated activation of lipolytic genes provides sufficient fatty acid substrates for the thermogenic process [33], indicating that the inguinal depot has formed a complete molecular pathway linking thermogenesis with energy supply under cold stress. In contrast, the cervical depot exhibited a marked response, characterized by an extremely significant increase in the mRNA expression level of *EBF2* alone following cold exposure. This seemingly discordant pattern may reflect a distinct regulatory strategy in the cervical depot, whereby the upregulation of *EBF2* represents an early cold-responsive signal [34] that is insufficient to initiate a full beiging program in the absence of coordinated activation of downstream coactivators such as *PGC1α*. Instead, this depot appears to favor energy storage, as evidenced by the downregulation of thermogenic gene expression and upregulation of *FABP4*, consistent with the physiological role of cervical adipose tissue as an insulating layer. The axillary depot exhibited moderate thermogenic activation, characterized by significant upregulation of *UCP3* at the protein level and extremely significant upregulation of mRNA expression of most beige marker genes. In contrast, the perirenal depot showed extremely significant upregulation of only *PGC1α* and *COX7A1* at the mRNA level, accompanied by significant downregulation of all other thermogenic and metabolic genes. These findings indicate that visceral depots possess limited beiging potential, consistent with previous rodent studies demonstrating weak browning capacity in perirenal adipose tissue [35].

Changes in the expression of adipogenic and lipid metabolism-related genes further support the depot-specific response patterns in the present study. In the inguinal depot, the synchronous upregulation of *FASN* and lipolytic genes indicates the presence of a dynamic balance between lipogenesis and lipolysis under cold stress, thereby providing sufficient substrates for thermogenesis while maintaining the metabolic activity of adipocytes. In contrast, both adipogenic and lipid metabolism-related genes were significantly downregulated in the perirenal depot, suggesting that cold stress inhibits its metabolic activity and promotes the maintenance of energy storage function. These differences are likely governed by depot-specific transcriptional regulatory networks. For example, *PPARγ*, a key regulator of adipogenesis and adipose tissue beiging [36,37], remained stably expressed in the inguinal depot, potentially providing the transcriptional basis necessary for beiging, whereas its marked downregulation in the perirenal depot may impede the beiging process.

The differentiation potential of preadipocytes is a key determinant of adipose tissue remodeling [38,39]. In vitro induction experiments in the present study demonstrated that preadipocytes derived from different depots of *Min pigs* exhibit marked heterogeneity in beiging potential, with inguinal preadipocytes displaying the strongest differentiation ability. It has been proposed that beige adipocytes originate from *PDGFR+* cell populations in white adipose progenitors and can be induced to undergo beiging in response to cold exposure, hormonal cues, or transcription factor modulation [40,41]. Oil Red O staining results revealed that inguinal preadipocytes formed tightly packed small lipid droplets following beiging induction, which is consistent with the cold stress-induced morphological characteristics in vivo. In contrast, preadipocytes derived from other depots only formed larger lipid droplets, suggesting that inguinal preadipocytes possess an innate tendency to generate small lipid droplets. Gene expression analysis further confirmed that following beiging induction, the expression ratios of all examined beige marker genes, adipogenic genes, and lipid metabolism-related genes in inguinal preadipocytes were significantly higher than those in the WAT group, forming a complete beige gene expression profile. In contrast, preadipocytes derived from the cervical, axillary, and perirenal depots showed significant increases in only some beige or metabolic gene ratios, indicating an incomplete beiging differentiation program. This result is highly consistent with the in vivo experimental conclusions, confirming that the strong beiging potential of the inguinal depot originates from the innate characteristics of its precursor cells rather than being solely induced by the local cold-stress microenvironment in vivo. Previous studies have shown that subcutaneous preadipocytes in pigs possess a greater beiging potential than visceral preadipocytes [42]. Based on these findings, the present study further identifies the inguinal depot as the principal site with high beiging potential. As a major interface between the body and the external environment, the pronounced beiging potential of inguinal preadipocytes may represent an important genetic adaption enabling *Min pigs* to cope with cold environments.

## 5. Conclusions

The present study demonstrates that cold stress induces depot-specific beiging remodeling of adipose tissue in *Min pigs*, with the inguinal depot representing the primary site exhibiting the highest beiging potential. The coordinated morphological and molecular responses observed in this depot constitute an important physiological basis for the enhanced cold tolerance of *Min pigs*. These findings provide new insights into the mechanisms underlying adaptive thermogenesis in large mammals and offer potential targets for environmental regulation and genetic improvement in pig production.

## 6. Limitations

Several limitations of the present study should be acknowledged. First, the sample size was relatively small (n = 5 per group) with uneven sex distribution. Nevertheless, sex was included as a balanced blocking factor instead of a primary experimental variable, and all male piglets were castrated to further reduce inter-individual hormonal variation. Second, although the single-litter experimental design compromises external generalizability, it was intentionally adopted to minimize inter-litter confounding factors, including maternal immunity, gut microbiota, and social-driven feeding competition, thereby improving internal validity for detecting treatment-driven physiological effects. Third, cold exposure was limited to a three-day acute challenge, which prevents the evaluation of long-term adaptive physiological responses. Fourth, infrared thermography outcomes are presented as representative images illustrating overall body-surface temperature changes; quantitative region-of-interest analysis targeting individual adipose depots could not be performed, as these targeted measurements were not systematically acquired in the original experimental setup. Even though thermography-related restraint was brief, fully standardized across all animals, and preceded by handling habituation, the non-anaesthetized restraint procedure may still trigger acute stress responses that could interfere with temperature readouts. Finally, our observations are derived exclusively from Min pigs and may not be extrapolated to other pig breeds or animal species. Future multi-litter investigations with larger animal cohorts are therefore required to validate these preliminary findings.

## Data Availability

The original contributions presented in this study are included in the article/Supplementary Materials. Further inquiries can be directed to the corresponding authors.

## Supplementary Materials

The following supporting information can be downloaded at: https://www.mdpi.com/article/10.3390/cells15181661/s1, Table S1: Sequences of primers used in this study.
